# Recombinant laccase from *Pediococcus acidilactici* CECT 5930 with ability to degrade tyramine

**DOI:** 10.1371/journal.pone.0186019

**Published:** 2017-10-11

**Authors:** Sara Callejón, Ramón Sendra, Sergi Ferrer, Isabel Pardo

**Affiliations:** 1 ENOLAB–Estructura de Recerca Interdisciplinar BioTecMed and Departament de Microbiologia i Ecologia. Universitat de València, c/ Dr. Moliner 50, Burjassot, Spain; 2 Departament de Bioquímica i Biologia Molecular. Universitat de València, c/ Dr. Moliner 50, Burjassot, Spain; Universidade Nova de Lisboa, PORTUGAL

## Abstract

Biogenic amines degradation by bacterial laccases is little known, so we have cloned and heterologously expressed, in *E*. *coli*, a new laccase from *Pediococcus acidilactici* CECT 5930 (Lpa5930), a lactic acid bacterium commonly found in foods able to degrade tyramine. The recombinant enzyme has been characterized by physical and biochemical assays. Here we report the optimization of expression and purification procedures of this laccase. DNA encoding sequence of laccase from *P*. *acidilactici* was amplified by PCR and cloned into the expression plasmid pET28a for induction by isopropyl-β-D-thiogalactoipyranoside. Protein expression was performed in *E*. *coli* BL21(DE3) harboring pGro7 plasmid expressing a chaperone folding assistant induced by arabinose. Purification was performed by column metal-chelating chromatography on Ni-NTA-agarose. The laccase enzyme obtained has an apparent molecular mass of ∼60 kDa, an optimum temperature activity toward 2,2′-azino-bis(3-ethylbenzothiazoline-6-sulfonic acid) (ABTS) of 28°C, and was quickly inactivated at temperatures higher than 70°C. The apparent K_m_ value for ABTS was 1.7 mM and the V_max_ obtained was 24 U/mg. In addition to ABTS, recombinant Lpa5930 laccase degraded the biogenic amine tyramine at pH 9.5 and pH 4.0 with or without ABTS as a mediator. Tyramine degradation by laccases could solve the problems generated in food due to the presence of this toxic compound.

## Introduction

*Pediococcus acidilactici* is a Gram-positive bacterium that can survive and grow in a wide range of pH, temperatures, and osmotic pressures, allowing them to inhabit the entire digestive tract of humans and different animals [1]. *P*. *acidilactici* is labeled as a *Generally Regarded As Safe* (GRAS) bacteria by the Food and Drug Administration (FDA). It can be found in fermented vegetables, fermented dairy products, and meat as a starter kit to add flavor and texture [2–4]. Its importance has increased since some strains of this species have been proposed as probiotic bacteria [5, 6]. *P*. *acidilactici* abilities include preventing the colonization and growth of pathogens, regulating and optimizing the function of the natural microorganisms. In addition, *P*. *acidilactici* strains also increase the nutritional value and digestibility of feed nutrients. Some strains of this species also can produce bacteriocins [7, 8]. Moreover, *P*. *acidilactici* has been used as microbial cell factories for bioconversion of lignocellulosic feedstocks. Boguta et al [9] and Ventorino et al [10] described diverse lactic acid bacteria (LAB) strains, *P*. *acidilactici* among them, capable of utilizing pentose sugars, as xylose and arabinose, and highly resistant towards common inhibitors from pretreated lignocellulosic biomass, such as furan derivatives, phenolic compounds and, weak acids. Recently we reported multicopper oxidases from LAB with a special new capacity toward biogenic amines (BA) [11]. BA are organic compounds of low molecular weight present naturally in animals, plants and microorganisms that possess biological activity [12]. These compounds are produced by decarboxylation of amino acids in foods containing free amino acids and which exhibit conditions that allow microbial and biochemical activity [12–14]. Thus, any fermented or raw product exposed to microbiological contamination may contain BA [15]. Consumption of foods containing high concentrations of BA is a concern for the consumer because they are related to health disorders [16]. Tyramine is associated with different symptoms such as difficulty breathing, hives, sweating, heat, burning mouth, palpitations, headache, nausea, diarrhea and hypertension [12, 13, 17, 18]. Some authors have reported relationships between poisoning outbreaks and tyramine concentrations of foods [12, 19].

Laccases (benzenediol:oxygen oxidoreductase, p-diphenol oxidase EC 1.10.3.2) are blue multicopper oxidases (MCO), abundantly present in many plants, fungal and bacterial species [20–22]. Laccases catalyze the monoelectronic oxidation of substrates at the expense of molecular oxygen and, generally, they had a broad range of substrates including phenols, such as methoxyphenols, polyphenols, and nonphenolic substrates, including aromatic amines, arylamines, anilines, thiols, and some cyanide complexes of metal. Laccases can expand their substrate range through the use of redox mediators, which are low molecular weight molecules that are able to shuttle electrons between laccases and target molecules that otherwise could not be oxidized [23]. Common artificial mediators are TEMPO (2,2,6,6-Tetramethylpiperidine-1-yl) oxyl; HBT (N-hydroxybenzotriazole), violuric acid, and ABTS 2,2’-azino-bis(3-ethylbenzthiazoline-6-sulfonic acid) [23]. Several naturally occurring compounds that mediate laccase activity have been identified as well [24, 25].

Laccase enzymes are being increasingly evaluated for a variety of biotechnological applications due to their broad substrate range [21]. In addition, these enzymes are used in green chemistry [26], for paper and pulp processing, in textile and petrochemical industries [27], in polymer synthesis and also in wine and beverage production. They have also used for bioremediation of contaminated soils and for detoxification of industrial effluents [28]. Laccases are also used as catalysts for the manufacture of anticancer drugs and even as ingredients in cosmetics, and they have also been applied to nanobiotechnology as biosensors or bioreporters [28, 29]. Laccases are catalogued as eco-friendly enzymes since they work with air and produce water as the only by-product [26]. Therefore, it seems an important innovation the study of laccases with novel capacities (e.g. BA degradation) to address new expectations about their potential use for biotechnological purposes.

To date, laccases have mostly been isolated and characterized from plants and fungi, and only fungal laccases are being currently used in biotechnological applications. Bacterial laccases, in contrast, have been less studied and applied to solve practical problems. So far only one report has described the use of a bacterial laccase in bioremediation of industrial wastes [30]. Only a few studies have been reported on the catalysis mechanism and stability at high pH and temperature of prokaryote laccases [31–33]. However, in principle, bacterial laccases could solve some of the limitations of fungal laccases in industrial and biotechnological processes. Bacterial laccases retain catalytic activity in a wider pH range [34, 35], exhibit higher thermal stability and are more tolerant towards organic solvents, high salt concentrations, and common laccase inhibitors than fungal laccases [27]. Likewise, bacterial laccases can be expressed with more feasibility than the fungal laccases in *E*. *coli*, although sometimes their heterologous expression results in enzymes that do not contain a full complement of copper ions.

Bacterial laccase was first reported by Givaudan et al. [36] from *Azospirillum lipoferum* isolated from rice rhizosphere. However, the most well-known and representative of this kind of enzymes is CotA from *Bacillus subtilis*, an endospore coat protein with high thermostability [37]. Bioinformatic studies on laccases have indicated that they are present in various high G + C gram-positive bacteria and also in α-, γ-, and ε-proteobacteria [38–40]. Laccase enzymes have been described in genomic sequences and analyzed in various other bacterial species such as *Escherichia coli* [41], several *Bacillus* species [31], *Thermus thermophilus* [42], diverse streptomycetes [43, 44] and one lactic acid bacterium strain *L*. *plantarum* J16 [45].

The aim of the present work was to clone and express the *P*. *acidilactici* CECT 5930 gene encoding the protein D2EK17, a putative MCO, and to characterize the expressed recombinant protein biochemically and physically including its capacity to react on BA.

## Materials and methods

### Materials

Usual laccase substrates [2,2-azino-bis(3-ethylbenzothiazoline-6-sulfonic acid) (ABTS) and 2,6-dimethoxyphenol (2,6-DMP)], amines histamine, tyramine, and putrescine, expression inducers isopropyl-β-D-thiogalactopyranoside (IPTG) and arabinose, antibiotics kanamycin, and chloramphenicol, and also standard proteins used for molecular weight determination were obtained from Sigma (Madrid, Spain). Nickel-chelating nitrilotriacetic acid (Ni-NTA) agarose was from Qiagen (Hilden, Germany). All other chemicals and reagents were of analytical grade.

### Strains, enzymes and plasmids

*P*. *acidilactici* CECT 5930 was obtained from Spanish Type Culture Collection (CECT) and routinely cultivated in MRS medium at 28°C.

*Escherichia coli* DH5α [F− Φ80*lac*ZΔM15 Δ(*lac*ZYA-*arg*F) U169 *deo*R *rec*A1 *end*A1 *hsd*R17 *sup*E44 λ-*thi*-1 *gyr*A96 *rel*A1] was used as a host for manipulation, amplification and propagation of the D2EK17 encoding gene. The employed overexpression vector was the plasmid pET-28a(+) (Novagen, Madison, WI, USA). *E*. *coli* BL21(DE3) [F- *dcm ompT hsdS* (rB^-^mB^-^) *gal* (DE3)] (Novagen), harboring also the pGro7 plasmid, was used as the host to express the gene of interest under the control of the T7 promoter. The plasmid pGro7, designed to enable efficient expression of multiple chaperones (groES- groEL) that work cooperatively in the protein folding process [46], was used for the coexpression of the protein D2EK17. *E*. *coli* cells were grown at 37°C in Luria–Bertani (LB) medium, while transformants, when appropriate, were grown in LB medium supplemented with kanamycin (50 μg/ml) or with kanamycin plus chloramphenicol (20 μg/ml).

Genomic DNA was purified by using the Ultra Clean® Microbial DNA Isolation Kit, plasmid DNA with the Ultra Clean™ 6 minute mini plasmid prep Kit and for purification of PCR products the Ultra Clean® PCR Clean-Up Kit was employed, all these kits from MoBio (Carlsbad, CA). DNA synthetic oligonucleotides were purchased from Eurofins MWG Operon (Ebersberg, Germany). Restriction enzymes were obtained from New England Biolabs (Beverly, MA), T4 DNA ligase was from Roche Diagnostics (Barcelona, Spain) and DNA polymerase and the corresponding 10X reaction buffer were from Invitrogen (La Jolla, CA).

### Cloning of gene encoding the D2EK17 protein and construction of the expression plasmid

To produce the recombinant enzyme tagged with a polyhistidine sequence at the N-terminus, the pET28a vector containing the nucleotide sequence encoding for the 6xHis tag was employed. The polyHis tag plus linkers added 20 amino acids to the recombinant protein at N-terminus.

The *P*. *acidilactici* CECT 5930 gene corresponding to the D2EK17 protein as a putative MCO was PCR amplified from purified genomic DNA with newly designed primers LacaPat1 5′- GAT***GCTAGC***ATGATTACAAAGTATCTATATGA-3′ (forward) and LacaPat2 5′- CCG***GGATCC***CTACATTTTATGGTCCATTGG-3′ (reverse). Recognition sites for *NheI* and *BamHI* endonucleases are indicated in bold italics. PCR was carried out using Taq DNA polymerase native (Invitrogen), in an Eppendorf thermocycler. The thermal profile setup was: initial denaturation (95°C for 5 min), 35 cycles of denaturation (94°C for 1 min), primer annealing (54°C for 1 min), and extension (72°C for 1 min). Finally, reactions were completed with 5-min elongation time at 72°C followed by cooling to 10°C. The PCR products were purified with the UltraClean® PCR Clean-Up Kit following the manufacturer’s instructions. The resulting purified fragment, containing the entire coding sequence plus linkers, and also the pET-28a plasmid vector were digested with *NheI* and *BamHI*. Following of the treatment of the linearized plasmid and after dephosphorylation with alkaline phosphatase, a ligation reaction was carried out with 100 ng of both D2EK17 and pET-28a vector and using T4 DNA ligase (2 U/ml) under standard experimental conditions, to obtain the plasmid pET-28a-Lpa5930. This recombinant plasmid was amplified in *E*. *coli* DH5α after transformation by electroporation. The pET28a-Lpa 5930 plasmid was then extracted, purified, and introduced, by heat shock transformation, into *E*. *coli* BL21(DE3) cells which already contained the pGro7 (TaKaRa) plasmid expressing GroES/GroEL chaperones. In parallel, transformation of *E*. *coli* BL21(DE3) without pGro7 was also performed. *E*. *coli* BL21(DE3) harboring the two plasmids, pET28a-Lpa 5930/pGro7, were grown at 37°C in LB medium containing kanamycin and chloramphenicol. Cells harboring only the pET28a-Lpa5930 plasmid were grown in medium with kanamycin.

### Optimization of bacterial expression of recombinant D2EK17 protein

In order to find appropriated conditions for an efficient production of the *P*. *acidilactici* CECT 5930 recombinant laccase, several parameters affecting culture growth and expression were tested, including post-induction temperature, regimen of stirring, presence of copper cation, and mediation of molecular chaperones expressed from a second co-existing plasmid.

*E*. *coli* transformed cells were grown in LB medium, supplemented with the corresponding antibiotics, under shaking conditions (250 rpm) at 37°C for about 16 h. Fifty ml of this preculture was employed to inoculate one liter of pre-warmed Terrific-Broth medium, containing the appropriated antibiotics (kanamycin and chloramphenicol) plus 2 mg/ml of arabinose, when needed to induce pGr07 plasmid. The culture was incubated at 28°C with orbital shaking at 250 rpm and, when the OD_600_ was around 0.6, IPTG was added to a final concentration of 1 mM to induce the expression of the recombinant laccase protein. Incubation of the culture was maintained under different conditions and time periods as described below. Two different final concentrations of copper (0.3 and 1 mM) in the growth medium were evaluated by adding CuCl_2_ at different stages of the cell culture. Two different post-induction temperatures, 37°C and 20°C, were checked [45]. After addition of IPTG cultures were incubated at 37°C (250 rpm) for 4 h before harvesting or at 20°C and shaking at 120 rpm for 4 h followed by an additional static incubation overnight (20°C) [47]. In all cases cells were finally harvested by centrifugation at 4°C at 13.500 rpm for 15 min (Beckman coulter Avanti J-E, JA10 rotor) and the resulting pellets were frozen and stored at -80°C.

### Recombinant laccase purification

Cell biomass from 1 liter culture was thawed and resuspended in 10 ml of lysis buffer (50 mM sodium phosphate, 300 mM NaCl, 20 mM imidazole, pH 8.0), containing 1 mg/ml lysozyme, 5 μg/ml DNase, 10 μg/ml RNase and, 1mM phenylmethylsulfonyl fluoride (PMSF). Cell suspension was incubated on ice for 30 min, and afterwards cells were disrupted mechanically with a similar volume of glass beads (106 μm) in a Mikro-Dismembrator (Sartorius) by setting 10 cycles of 40s. Cell debris was removed by centrifugation at 13,500 rpm for 15 min at 4°C (Multifuge 1 S-R, Heraeus). The supernatant was collected and loaded onto a Ni^2+^-NTA–agarose column (0.8 x 2.5 cm), previously equilibrated with equilibration buffer (50 mM sodium phosphate, 300 mM NaCl, 20 mM imidazole, pH 8.0). Non-retained proteins were washed out with five column volumes of equilibration buffer. The retained protein was eluted with elution buffer (equilibration buffer containing 250 mM imidazole). Fractions of 0.5 ml were collected, tested for relative protein concentration by monitoring A_280_, and examined for laccase activity, using ABTS as substrate, in a liquid assay (described below). Fractions displaying activity were pooled and dialyzed overnight against 50 mM sodium phosphate, pH 7.4, containing 1 mM CuCl_2_ and 0.05% Tween 20. The content and complexity of proteins present in the chromatographic fractions and in the crude extract were analyzed by sodium dodecyl sulfate–polyacrylamide gel electrophoresis (SDS-PAGE) to check the degree of purity achieved in the purification procedure. Enzyme activity throughout the process of enzyme extraction and purification was also evaluated by the in-gel assay described by Callejón et al. [11]. Protein concentrations of all samples were measured by bicinchoninic acid (BCA) assay with bovine serum albumin as protein standard [48].

### Laccase activity assays

Laccase activity was assessed by both in-gel and liquid assays [45]. Briefly, in-gel assays were performed after protein separation by native 8% polyacrylamide gel electrophoresis [11]. Gels were subsequently incubated at room temperature in 0.1 M sodium acetate buffer, pH 4.0, containing 10 mM 2,6-DMP for 5 min. Then, the gel was incubated with 1 mM CuSO_4_ in the same buffer where the laccase activity was visualized by the presence of an orange-colored band. Liquid assays were carried out by mixing aliquots from the different fractions with 100 μL of buffer (50 mM of acetate buffer, pH 4.0, with 5 mM ABTS as a substrate), and incubation at room temperature. Oxidation of ABTS was determined by absorbance increase in microplate wells at 420 nm (ε_420_ = 36,000 M^−1^ cm^−1^) in a 96-well microplate reader.

### Biochemical characterization of *P*. *acidilactici* D2EK17 enzyme

Pure recombinant *P*. *acidilactici* D2EK17 protein, in 50 mM potassium phosphate buffer, pH 6.5, was employed to acquire UV–visible absorption spectra (300–800 nm) by using a Beckman Coulter DU® 800 UV/Vis spectrophotometer.

The relative molecular weight of the enzyme polypeptide was determined by comparison with molecular weight markers in a SDS-8% PAGE using the LMW-SDS Marker Kit (GE Healthcare Life Sciences).

The following characterization experiments were performed by the liquid assay described above, using 0.7 μg of the recombinant enzyme for each assay point. To determine the pH dependence of the recombinant laccase towards ABTS (5 mM) as a substrate, the standard liquid assay was employed by using 50 mM citrate-phosphate buffer to cover a range from 2.5 to 8.0. Oxidation of ABTS was measured by the absorbance increase at 420 nm (ε_420_ = 36,000 M^−1^ cm^−1^) in microplate wells.

The effect of temperature on D2EK17 laccase activity in the range 4 to 65°C was studied by measuring ABTS oxidation in the standard liquid assay. The reaction mixtures were set at the appropriate temperature for 10 min previously to the enzyme addition. For thermal stability tests, aliquots of the enzyme solution, in 50 mM acetate buffer, pH 4.0, were pre-incubated at different temperatures at 28, 35, 40, 45, 60, 75, and 85°C for 10 min and then, after cooling of the samples, the residual activity was determined under standard liquid ABTS assay conditions.

To determine kinetic parameters V_max_ and K_m_ of the recombinant laccase for ABTS it was incubated with increasing concentrations of ABTS (0.1–50 mM) in acetate buffer, pH 4.0, at 28°C. Reactions in triplicate were initiated by the addition of the enzyme and the absorbance increases at 420 nm were recorded for 10 min. The initial rates were deduced from the slope of plots of A_420_ versus time. Data were fitted to the Michaelis–Menten equation to determine the V_max_ and K_m_ values. One enzyme activity unit (U) was defined as the amount of enzyme that oxidizes 1 μmol of substrate/min in the standard liquid assay.

To demonstrate that copper chloride does not inhibit the activity of the enzyme, this activity was tested in the presence of CuCl_2_ and CuSO_4_ at 0.1 and 1mM.

The effect of metal-chelating agents, such as bipyridyl, phenanthroline, SDS and EDTA, in addition to other compounds that potentially could affect the oxidizing activity of the enzyme, such as N-(3-dimethylaminopropyl)-N′-ethylcarbodiimide (EDC) and semicarbazide, was assessed by incubating the enzyme solution for 5 min at 28°C in the presence of 100 μM of these compounds. After this preincubation, the reaction was initiated by the addition of the substrate ABTS and the residual activity was determined by the liquid assay. Enzyme activity in the absence of any agent was considered as 100% relative activity (control).

### Oxidation of amines by *P*. *acidilactici* D2EK17 laccase

The amine-oxidizing ability of the recombinant *P*. *acidilactici* laccase, under different conditions, was analyzed by quantification of remaining amine in the reaction mixtures after incubation of the enzyme with histamine, tyramine or putrescine (150 mg/L) [45]. Different enzyme activity assay conditions, including enzyme concentration (7, 14, 28, 56 and 140 μg/assay), the presence and concentration of ABTS (0–10 mM) and HBT (5 and 10 mM) as mediators, the presence of CuCl_2_ (100 μM), temperature (range 4–45°C), pH (2.5–10.5) and agitation regimen were assessed using tyramine as a substrate. Incubations were carried out with 100 μL of reaction volume in 50 mM acetate buffer, pH 4.0, except for pH dependence assays that was evaluated by using 50 mM citrate-phosphate (pH range from 2.5 to 8.0) and 50 mM carbonate-NaOH (range from 9.0 to 10.5).

Reaction solutions were incubated at 28°C for 24 hours, after which remaining amine concentration was determined by LC-FLD (see below). As negative controls and under identical conditions, mixtures without enzyme or with heat-inactivated enzyme were used.

Amines were quantified by reverse-phase LC-FLD as previously described by Callejón et al [45]. Identification of compounds was performed by comparing retention times of known standards of tyramine, histamine, and putrescine (Sigma). Fluorometric detection was done with the excitation and emission wavelengths at 340 and 430, respectively. The amine concentrations were determined using the peak areas relative to the area of internal standard. The percentage of amine degradation was calculated respect to a negative control (incubation in the absence of enzyme), and used to deduce the relative activity as a percentage of the highest degradation value obtained in each experiment.

### Statistical analyses

All data are represented as the mean ± SE. Group means were compared using one-way ANOVA followed by Duncan’s multiple range tests to identify differences among groups when appropriate. All analyses were carried out using IBM SPSS Statistics 19 (SPSS Inc., Chicago, IL, USA). Percentage data were normalized by arcsine transformation prior to the analysis.

## Results

### Isolation and sequence analysis of the D2EK17 gene from *P*. *acidilactici* CECT 5930

**Fig 1:**
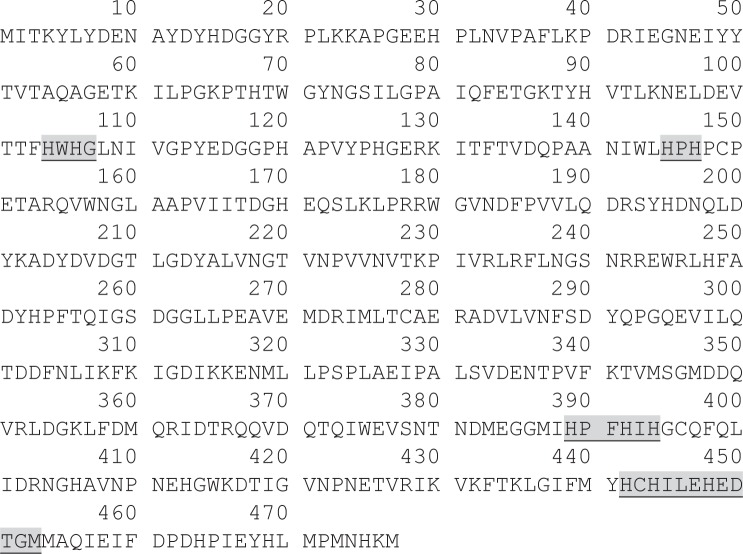
Amino acid sequence of *P*. *acidilactici* CECT 5930 protein homologue of D2EK17. Shaded and underlined are the motifs corresponding to the four copper ligands which are strictly conserved in MCOs (conserved sequence of these motifs are, successively: HXHG, HXH, HXXHXH and HCHXXXHXXXXM/L/F).

Previously we purified a protein associated to an oxidizing activity of canonical MCO substrates and different amines from *P*. *acidilactici* CECT 5930, which was identified as a homologue of D2EK17 protein from *P*. *acidilactici* 7_4 [11]. Currently, in the UniProtKB database, D2EK17 is described as a putative multicopper oxidase. As described in Material and Methods section, two new primers, LacaPat1/LacaPat2, were designed from the D2EK17 *P*. *acidilactici* 7_4 gene, published as a part of its complete genome sequence (NZ_ACXB00000000), and used to amplify, clone, and characterize the D2EK17 homologous gene from *P*. *acidilactici* CECT 5930. DNA sequence analysis of the complete gene (1434 bp), with Mega 5 software, showed four short sequences encoding for the four strictly conserved copper ligand motifs which are characteristic of the MCO family [49] (Fig 1).

In addition, multiple sequence alignment, performed with ClustalOmega software (http://www.ebi.ac.uk/Tools/msa/clustalo/), indicated that the MCO D2EK17-homologous protein from *P*. *acidilactici* CECT 5930 showed a 21.68% amino acid sequence identity with the laccase from the common mushroom *Trametes versicolor* (UniProt ID: Q96UK8). Likewise, the comparison with other known prokaryotic laccases showed 30.61% identity with *Bacillus* sp. CotA, and 38.26% with *E*. *coli* CueO (UniProt ID: I3RYX9 and P36649, respectively). Furthermore it has an identity of 58.28% with *L*. *plantarum* JDM1 SufI cell division protein (UniProt ID: A0A023M894), the homologous of the *L*. *plantarum* J16 CECT 8944 SufI protein recently identified as laccase by us [45].

### Cloning and improvement of the recombinant laccase expression

*P*. *acidilactici* CECT 5930 D2EK17 laccase gene was cloned into the pET28a expression plasmid and the resulting construction (pET28a-Lpa 5930) transformed into *E*. *coli* BL21(DE3) cells that harbor, or not, a second plasmid (pGro7) for the co-expression of a molecular chaperone system. When the *E*. *coli* BL21(DE3) cells did not contain the chaperone folding assistant plasmid, the expressed protein did not exhibit enzyme activity on the standard laccase substrate 2,6-DMP. Only a low enzyme activity was detected when the expression was carried out on cells that also contain the pGro7 vector, and under standard conditions at 37°C. The addition of up to 1 mM CuCl_2_ to the already expressed protein or dialysis against a buffer containing 0.3 mM CuCl_2_, did not result in an additional increase of enzyme activity (results not shown). On the contrary, a high 2,6-DMP oxidizing activity was obtained, in an in-gel assay, when the induction was made in a medium containing CuCl_2_, at 37°C and agitation at 200 rpm (data not shown), indicating the requirement of Cu^2+^ in the culture medium for the proper expression of the recombinant MCO in active state. Previous studies have indicated that under microaeration conditions, as those obtained in static cultures, and temperatures lower than those commonly employed in the bacterial production of recombinant proteins, enhances the expression level and the specific activity of recombinant laccases are considerably increased [45, 47, 50]. Thus, we have also found an important increase in the recovering of soluble and active recombinant *P*. *acidilactici* CECT 5930 enzyme. After testing additional different conditions, the most appropriate we found, to attain the best results on the protein expression and enzyme activity, were fairly similar to those previously described for the production of recombinant *L*. *plantarum* J16 CECT 8944 [45]. In brief, *E*. *coli* BL21(DE3) cells containing both plasmids, the pET-28a-Lpa5930 and pGro7 plasmid, are grown at 37°C in LB medium in the presence of 1 mM CuCl_2_ and stirring at 200 rpm up to reach the exponential phase of growth. After adding inductor (IPTG 1 mM) the culture is incubated at 20°C with stirring at 120 rpm for four hours and then the stirring is turned off and the incubation is extended overnight at the same temperature. Seemingly, the microaerobic state, resulting in the static incubation, enhances the intracellular accumulation of copper cation, facilitating the proper copper loading and folding of the protein, and leading to recover a high yield of soluble active recombinant laccase [47, 51, 52].

### Purification of the recombinant *P*. *acidilactici* CECT 5930 laccase

**Fig 2:**
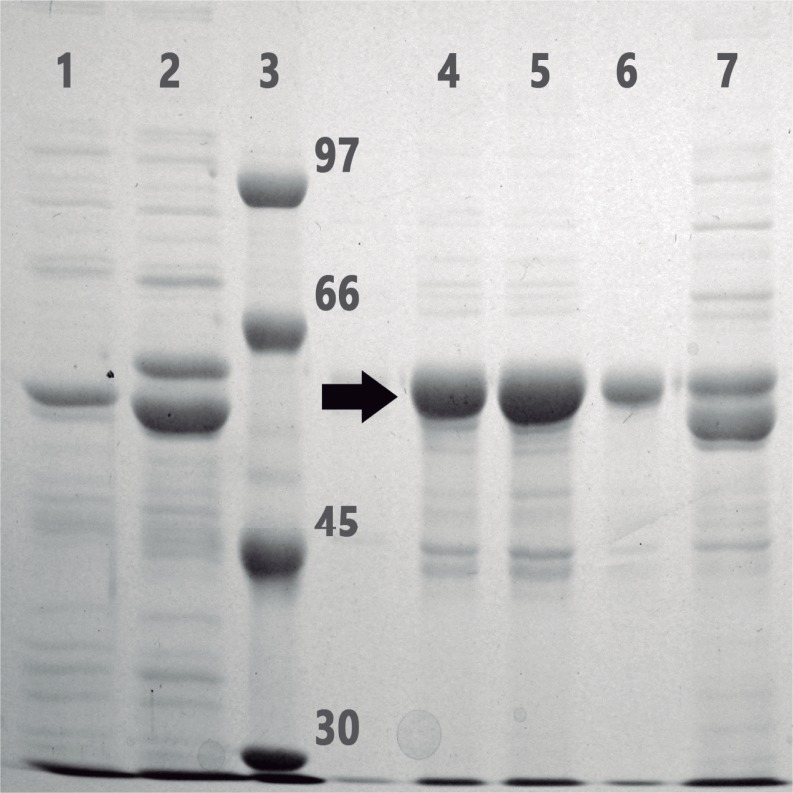
Coomassie–blue stained gel after SDS-8% PAGE of different fractions during the purification procedure of recombinant *P*. *acidilactici* CECT 5930 laccase. Lane 1, whole-cell extract from non-induced cells; lane 2, whole-cell extract from post-induction cells; lane 3, protein markers, with the molecular weights indicated on the right (kDa); lanes 4–6, successive fractions of the elution from the metal-chelating chromatography on Ni^2+^-NTA-agarose; lane 7, crude extract corresponding to applied sample on the chromatography column. Arrow marks recombinant protein.

For the preparative purification of the recombinant enzyme in a soluble and active form, a crude cell extract prepared by bead-beating lysis of cells from a liter culture subjected to the conditions described above was obtained. The densitometry analysis (with the ImageJ software) of the Coomasie-blue stained gel, after a SDS-PAGE, indicated that the amount of post-induction overexpressed recombinant laccase protein represented around 9% of total protein (Fig 2). Recombinant *P*. *acidilactici* laccase that contains a polyhistidine N-terminal extension was purified from the crude extract by metal-chelating chromatography on Ni^2+^-NTA agarose. Laccase activity in the different column fractions was revealed by in-gel assay with 2,6-DMP as a substrate, and their protein content was analyzed by SDS-PAGE (Fig 2). Those chromatographic fractions with the highest levels of enzyme activity, and that also showed an intense blue color, were pooled together and after dialysis for desalting, frozen and stored at -80°C. About 14 mg of pure recombinant protein was recovered from one liter of cell culture. The specific activity of purified recombinant laccase on ABTS, determined by the standard liquid assay, was around 24, 1 U/mg.

### Properties of the recombinant *P*. *acidilactici* laccase

Electrophoretic analysis of the purified recombinant enzyme on SDS-polyacrylamide gel (Fig 2) revealed an apparent molecular weight of around 60 kDa. As the recombinant protein contains an artificial 2,5 kDa N-terminal extension, including the (6xHis) tag plus a short amino acid linker stretch, the molecular weight of the catalytic polypeptide chain of *P*. *acidilactici* laccase is very near to that inferred from the cloned gene sequence (54.36 kDa).

**Fig 3:**
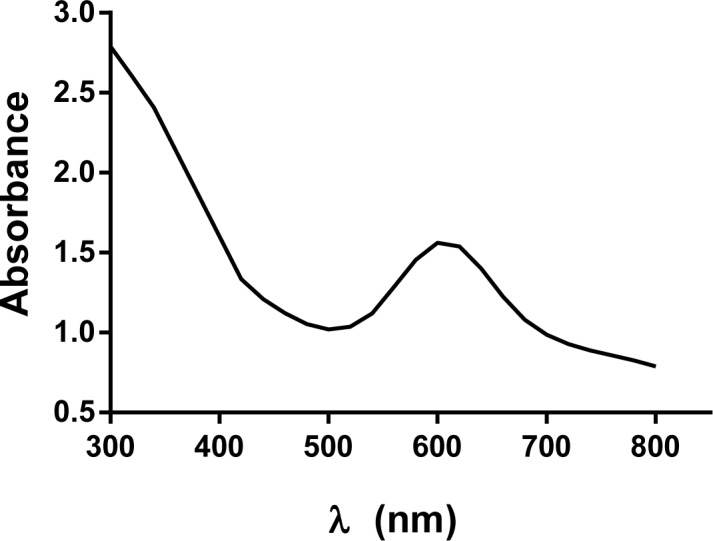
UV-Visible spectrum of purified recombinant *P*. *acidilactici* CECT 5930 laccase. Protein was dissolved in 50 mM sodium phosphate, pH 6.5 buffer at a concentration of 14 mg/ml. Only the range from 300 to 800 nm is shown.

As it is characteristic of the laccase enzymes, the purified recombinant protein showed an intense blue color, and exhibited a maximum peak at 590 nm in the UV–visible absorption spectrum (Fig 3), which corresponds to the T1 copper center of the MCOs. The final absorbance 280/600 ratio of the purified enzyme was 1.9, indicating a good purification level [51].

**Fig 4:**
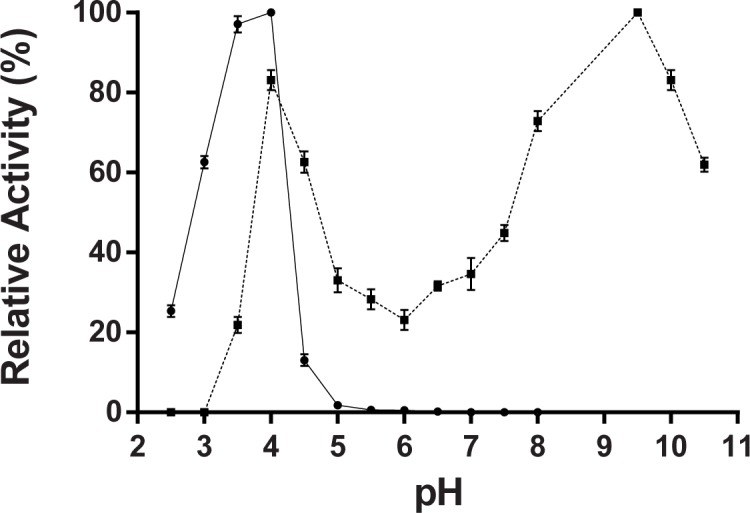
pH effect on activity of the recombinant *P*. *acidilactici* enzyme toward ABTS (*solid line*); and tyramine (*dashed line*) as substrates. Citrate-phosphate buffer (pH range 2.5–8.0) and carbonate-NaOH buffer (pH range 9.0–10.5) were used at concentration of 50 mM. Enzyme activity is plotted as percentage relative to the maximum value for each substrate (% relative activity). With tyramine as a substrate, the 100% of relative activity means a 43% of tyramine degradation in the reaction mixture, determined by reverse-phase LC-FLD, under the assay conditions described in Material and Methods section. The values are means ± standard deviations of triplicate assays.

**Fig 5:**
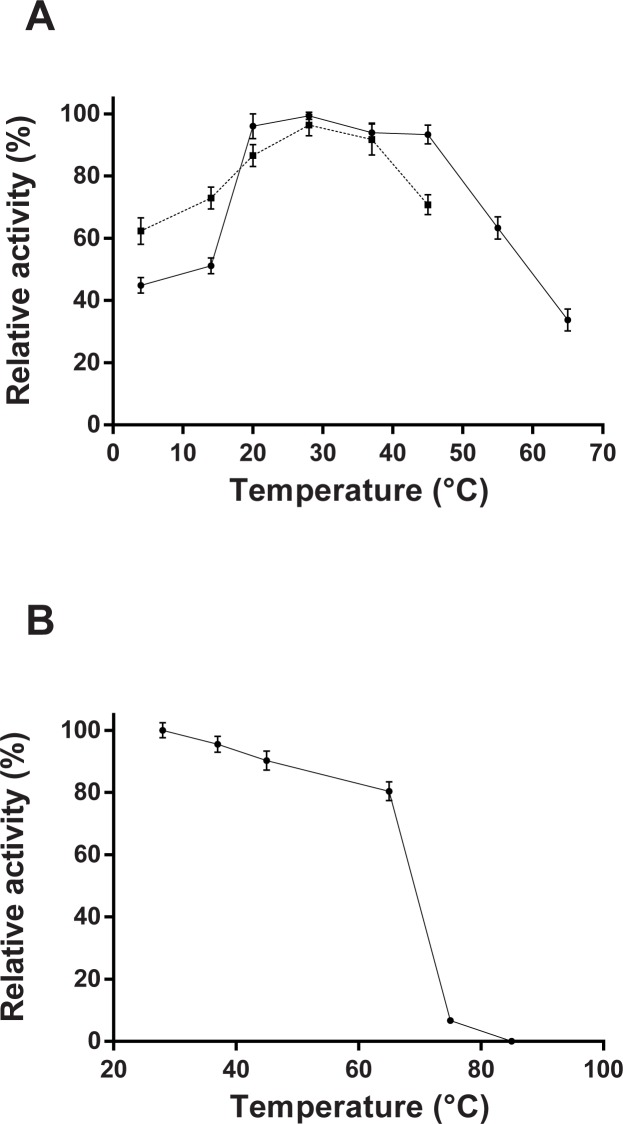
(A) Temperature dependence of recombinant *P*. *acidilactici* enzyme determined by incubation of the reaction mixtures, at the indicated temperatures, and using the standard liquid assay with ABTS (*solid line*) and tyramine (*dashed line*). Enzyme activity is plotted as a percentage relative to the maximum value (% relative activity). When tyramine was the substrate the 100% of relative activity represents a 70% of tyramine degradation in the assay conditions described in Material and Methods section. (B) Thermal stability analyzed by preincubation of the enzyme solution at the showed temperatures for 10 min before being reequilibrated to 28°C and employed for the standard liquid assay with ABTS as a substrate. Residual enzymatic activity, relative to the maximum value (28°C), is graphed against the pre-treatment temperature. All values are means ± standard deviations for triplicate assays.

The pH dependence analysis of the *P*. *acidilactici* laccase activity towards ABTS as a substrate revealed a sharp peak with a maximum around pH 4.0 (Fig 4). No significant ABTS-oxidizing activity was detected above pH 5.5. The optimal temperature for recombinant enzyme, on the canonical laccase substrate ABTS, was 28°C, although the enzyme retained a high level of activity in a wide temperature range, from 4 to 65°C (Fig 5A). Heat inactivation experiments revealed that *P*. *acidilactici* laccase is a relatively thermostable enzyme since the recombinant enzyme retained more than 80% of the activity after a pre-treatment at 60°C for 10 min (Fig 5B). Temperatures higher than 85°C, however, irreversibly inactivated the recombinant enzyme (Fig 5B).

Kinetic analysis of *P*. *acidilactici* laccase with ABTS as a substrate showed a hyperbolic dependence of the reaction rate on substrate concentration, and provided the K_m_ and V_max_ values of 1.7 mM and 24 U/mg, respectively.

Whereas the presence of copper is mandatory for the activity of the enzyme (supplementary figure S1 Fig), no significant differences was observed in the presence of 0.1 and 1.0 mM of the copper salts, CuCl_2_ and CuSO_4_. This demonstrates that Cl^-^ ions have not an inhibitory effect on laccase activity.

**Fig 6:**
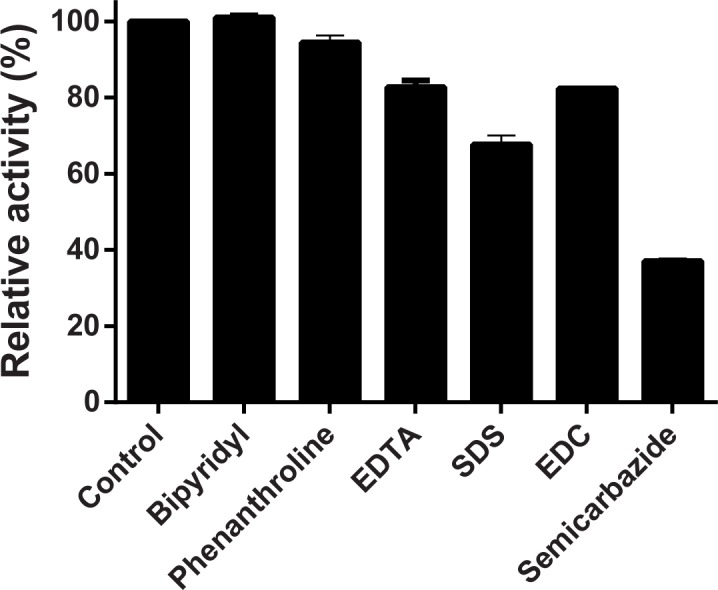
Effect of potential inhibitors of *P*. *acidilactici* laccase. Liquid assays using ABTS as a substrate were carried out in the presence of 100 μM of each of the indicated compounds. Control was an enzyme assay in the absence of inhibitor and is taken as 100% of activity. Results express the remaining activity as percentage relative to the control. Values are means ± standard deviations of triplicate assays.

The effect of different potential inhibitors on *P*. *acidilactici* laccase activity is shown in Fig 6. EDTA and SDS at 100 μM diminished the activity by 18% and 33%, respectively, although the other two tested metal-chelating agents, bipyridyl and phenanthroline, did not significantly inhibit the enzyme. The carboxyl modifier 1-ethyl-3-(3-dimethylaminopropyl) carbodiimide (EDC) produced only a weak inhibition around 18%. Finally, semicarbazide, a carbonyl-modifying reagent which inhibits a subgroup of copper-dependent amine oxidases, decreased a 63.3% of the ABTS-oxidizing activity of the recombinant enzyme.

### Biogenic amine-oxidizing activity of *P*. *acidilactici* laccase

We previously detected in *P*. *acidilactici* CECT 5930 an enzyme with the capacity to degrade BA, which was identified as MCO, and in this work, it has been cloned and expressed. So, we have deepened analyzing the recombinant enzyme ability to oxidize BA. Our analysis have focused on those BA found most frequently in foods, tyramine, histamine and putrescine. Amine degradation by the recombinant laccase was monitored by reverse-phase LC-FLD measuring of the remaining amine in the post-incubation reaction mixture [45] (supplementary figure S2 Fig). Firstly, we observed a linear dependence of tyramine degradation using between 7–56 μg of recombinant protein per assay point. Thus, all subsequent characterization experiments, with tyramine as substrate, were performed with 14 μg of recombinant enzyme in each reaction mixture. The inclusion of 100 μM CuCl_2_ to the reaction medium produced an enhancement in the tyramine oxidation, similarly to our results with the *L*. *plantarum* laccase [45], although higher concentrations of CuCl_2_ did not result in further increases in the amine degradation (not shown).

As it can be seen in Fig 4, the oxidizing activity of *P*. *acidilactici* laccase on the tyramine was maintained in a rather wide pH range, from 3.0 to near 11.0, although two clear maxima were observed, at pH 4.0 and pH 9.5, in which the relative activity of the enzyme was 82% and 100%, corresponding to a 35.6 and 43% of tyramine degradation respectively. No tyramine degradation was observed at any pH in the absence of enzyme or with heat inactivated enzyme in the control assays. It shows that the pH effect on the enzyme activity acting on tyramine is very different from ABTS, indicating that it depends on the type of substrate, which is a typical feature of laccases. Regarding the temperature dependence for tyramine degradation, it was found to be similar that for the canonical laccase substrate, ABTS (Fig 5A), with an optimum temperature at 28°C. Under the assay conditions employed, the recombinant *P*. *acidilactici* laccase retained up to 70% of the tyramine-degrading activity, regarding a 100% of relative activity at 28°C. (Fig 5A).

**Fig 7:**
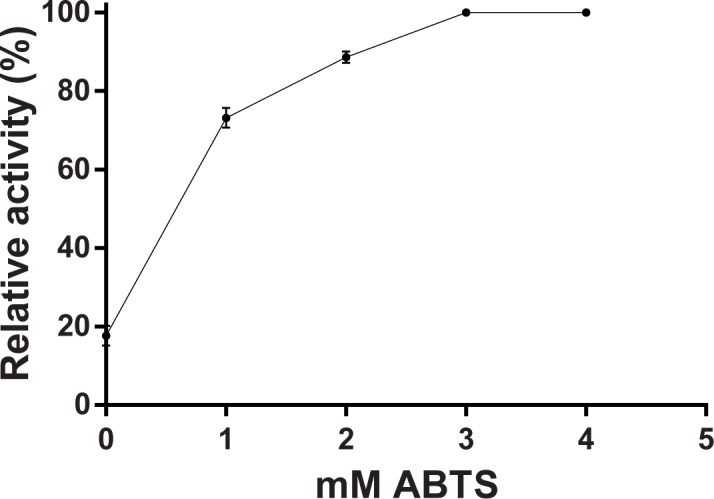
Function of the ABTS as redox mediator on tyramine oxidation by recombinant *P*. *acidilactici* laccase. Tyramine concentration was quantified by reverse-phase LC-FLD after incubation with recombinant laccase and different concentrations of ABTS for 24 at 28°C. The percentages of tyramine degradation were calculated regarding a control without enzyme. Activity is expressed as percentage relative to the maximum, where a 100% means a 75.2% of tyramine degradation under these assay conditions. The values are means ± standard deviations for triplicate assays.

As shown in Fig 7, the addition of ABTS to the incubation mixture increased the tyramine degradation by the recombinant *P*. *acidilactici* laccase, indicating that ABTS is acting as a mediator. The percentage of the amine degradation augmented with the ABTS concentration up to achieve a 75.2% degradation (100% relative activity) as can be seen in Fig 7. The control assays in the absence of enzyme, as described in Material and Methods section, indicated that ABTS by itself did not produce tyramine degradation.

The oxidizing potential of other BAs, histamine and putrescine, by the recombinant enzyme was initially tested using the assay conditions found to be optimal for tyramine, and described above (100 μL sodium acetate buffer 50 mM, pH 4.0, 150 mg/L BA, in the presence of 1 mM ABTS and 100 μM CuCl_2_, 14 μg enzyme, and incubation for 24 h at 28°C). However, results showed that recombinant *P*. *acidilactici* laccase was unable to degrade histamine and putrescine. We neither detected oxidizing activity on these other BAs at pH 9.5 or after the addition of up 5 mM ABTS. It neither was observed degradation of histamine and putrescine when another mediator, HBT, at several concentrations, was tested. HBT besides did not affect the capacity of recombinant laccase to degrade tyramine.

## Discussion

Amino acid sequence of the *P*. *acidilactici* CECT 5930 laccase clearly shows that the motifs for Cu ligands type 1 site and trinuclear cluster are present, and located in the same relative positions as found in other bacterial laccases. Furthermore, according to the Laccase Engineering Database (https://lcced.biocatnet.de/), *P*. *acidilactici* laccase belongs to the SUBfamily J with homologues of CueO from *E*. *coli*. Along with these data, its intense blue color, that characterize all “blue” copper oxidases, the 280/600 nm absorbance ratio in the typical range for bacterial laccases [51], and its oxidizing catalytic activity of representative substrates of laccase, like ABTS and 2,6-DMP, ratifies that it is a genuine multicopper oxidase, subtype laccase.

The expression of full functional laccases is confronted with the problem of the insufficient Cu loading of the recombinant protein. Although, *E*. *coli* cells prevent intracellular accumulation of toxic copper with inducible efflux systems [52], several strategies have been applied to increase copper loading. As cells grown under anoxic conditions do not induce Cu efflux systems to the same extent, the simply introducing of a static cultivation step after induction resulted in fully copper loaded and active recombinant CotA laccase from *Bacillus subtilis* [47] and also from *Bacillus pumilus* [33]. Nonetheless, microaerobic expression led to reduced growth rates which limited the performance of this condition for large scale production of laccases.

Moreover, the inclusion of copper salts to the expression medium can increase the specific copper loading [53]. In addition, depleted copper centers can be reconstituted after expression and purification by incubation or dialysis at high copper concentrations [42, 54], although these treatments not always lead to the recovering of a full active laccase enzyme [45, 47]. Gunne et al. [55] applied two complementary resources for increasing the copper content of recombinant CotA from *B*. *licheniformis*: a knock-down of the host copper detoxification system, and a helping coexpression of a chaperone. Therefore, not only intracellular copper ion concentration, but also the assistance of an appropriate chaperone influences copper ion insertion into CotA laccase. The presence of the coexpressed protein chaperones must facilitate copper incorporation to the clusters during the folding process or alternatively, also must increase soluble recombinant polypeptide available for the copper integration.

In this work, we managed to increase the expression of recombinant *P*. *acidilactici* laccase in *E*.*coli* in a catalytically active form, diminishing temperature during induction, by the inclusion of copper to the expression medium, and maintaining a low aerobic level by removing stirring, similarly as already reported for *L*. *plantarum* J16 CECT 8944 laccase [45], but in the present case the coexpression of chaperones it was needed to prevent misfolding. It is assumed that under these conditions, on one hand the exogenously added copper and the microaeobic medium contribute to an appropriate intracellular accumulation of copper, and on the other hand, the decrease of the post-induction culture temperature enhances the level of the recombinant protein expression [47, 50, 53].

Our biochemical characterization of the *P*. *acidilactici* laccase has shown that the enzyme has a molecular weight around 60 kDa, which is in the range found for several other bacterial laccases, most of which fall between 50 and 100 kDa [40]. Besides, as many other laccases from bacteria and fungi [56, 57], the optimum pH of *P*. *acidilactici* recombinant laccase, with ABTS as a substrate, is 4,0; and likewise also the activity profile against pH is dependent on the nature of substrate, showing a maximum around 3.5–4.0 with ABTS, and two maxima at 4.0 and 9.5 with tyramine. The optimum temperature for *P*. *acidilactici* enzyme is around 28°C, which is lower than those of other several bacterial laccases, such as CotA of *Bacillus* species (55–75°C) [33, 47, 53] or CueO from *E*. *coli* (55°C) [41]. Nevertheless, its thermostability is similar to *E*. *coli* CueO [41] and *L*. *plantarum* J16 laccase [45], maintaining at least 80% of activity after 10 min incubation at 65°C, but lower than *B*. *subtilis* CotA [47] and *B*. *tequilensis* laccase [58], which retained more than 50% of activity after a long incubation (>100 min) at temperatures above 70°C. Regarding the kinetic analysis of the recombinant *P*. *acidilactici* laccase, our results provided a K_m_ value of 1.7 mM for ABTS, somewhat higher than those described for laccases from several species of *Bacillus* and other bacteria [33, 45, 53, 59], but fairly similar to that of *L*. *plantarum* J16 [45].

Strikingly, the *P*. *acidilactici* laccase was not inhibited by the metal-chelating agents bipyridyl and phenathroline (at 100 μM), but it was by EDTA and SDS, as expected given prior results with diverse laccases from different organisms [43]. Likewise, we previously observed a strong inhibition of the *L*. *plantarum* J16 laccase by EDC [33, 45, 53, 59], however in this work the recombinant *P*. *acidilactici* laccase was only marginally inhibited by this carboxyl-group modifying agent. Therefore, the apparent involvement of a carboxyl group in the catalysis, as has been already proposed for some fungal laccases [60] seems to be less important in the *P*. *acidilactici* laccase. Conversely, the inhibitory effect of semicarbazide, a carbonyl-modifying agent that inhibits a subgroup of copper-dependent amine oxidases, on the recombinant *P*. *acidilactici* enzyme was higher than on *L*. *plantarum* J16 laccase [33, 45, 53, 59]. Inhibition by semicarbazide has been also described for the laccase CueO [41].

The ascertaining that laccase enzymes, including the commercial laccase from the fungus *Trametes versicolor* [11], are able to degrade biogenic amines in foods underscores a new interest on laccases for their potential applications in food biotechnology. However, the studies about the BA-oxidizing capability of prokaryotic and eukaryotic laccases are still scarce. Recently we have identified, cloned and characterized the SufI protein of *L*. *plantarum* J16, a LAB isolated from wine but that also is frequently found in diverse foods, as laccase bearing the ability to degrade the BA tyramine, and also, although in a lesser extent, histamine and putrescine [45]. In the present work, we have found that recombinant *P*. *acidilactici* laccase is also able to degrade tyramine but, contrary to *L*. *plantarum* enzyme, apparently not histamine nor putrescine. Possibly the suitability of tyramine being oxidized by the recombinant laccase is in the phenolic nature of this substrate [33]. Besides, we have found out that the activity of recombinant *P*. *acidilactici* laccase toward tyramine can be significantly enhanced by presence of ABTS acting as redox mediator. Overall the *P*. *acidilactici* laccase differs from the *L*. *plantarum* J16 laccase in terms of its physical and biochemical properties, such as response to inhibitory substances, optimal temperature, thermostability and kinetic parameters; however both enzymes exhibit a very similar oxidizing capability towards tyramine.

## Supporting information

S1 Fig Effect of Cl^-^ ions on the *P*. *acidilactici* laccase activity. The enzyme activity was measured as increase in absorbance at 420 nm against 0.1 and 1.0 mM CuCl_2_ and CuSO_4_ using ABTS as a substrate.
(EPS)

S2 Fig Tyramine degradation analysis by LC-FLD. Tyramine (150 mg/L) was incubated with 2 μL of *P*. *acidilactici* CECT 5930 recombinant enzyme (28 μg) for 24 h, as described in Material and Methods section. Then, remaining tyramine concentration was determined by LC-FLD. The graph shows the retention time on the x-axis *versus* fluorescence units (LU) recorded by the detector (y-axis). Only the chromatographic profile region corresponding to the tyramine peak is shown. Note the peak area corresponding to incubation with recombinant enzyme (red) is approximately 35% lower than the control (blue). As negative controls, an identical mixture without enzyme (control 1), and with heat inactivated enzyme (control 2) were used. Since both controls gave the same peak area, only one is shown (blue).
(PDF)
