# Supplementary material for: Recombinant laccase from Pediococcus acidilactici CECT 5930 with ability to degrade tyramine
Source: PLoS One. 2017 Oct 11;12(10):e0186019. doi: 10.1371/journal.pone.0186019 (PMC5636118; doi:10.1371/journal.pone.0186019)

```
Print of window 38: Current Chromatogram(s)
Data File  : C:\CHEM32\1\DATA\AB 2013-01-22 11-33-12\1AE-0501.D
Sample Name : CTCu
=====================================================================
Acq. Operator   :                              Seq. Line :    5
Acq. Instrument : Instrument 1                  Location : P1-A-05
Injection Date  : 22/01/2013 13:16:14                Inj :    1
                                              Inj Volume : Inj prog
Acq. Method     : C:\CHEM32\1\DATA\AB 2013-01-22 11-33-12\AB-PFP.M
Last changed    : 22/01/2013 9:35:26
Analysis Method : C:\CHEM32\1\METHODS\BA-FIN.M
Last changed    : 07/06/2017 16:33:49
Method Info     :
```

Current Chromatogram(s)

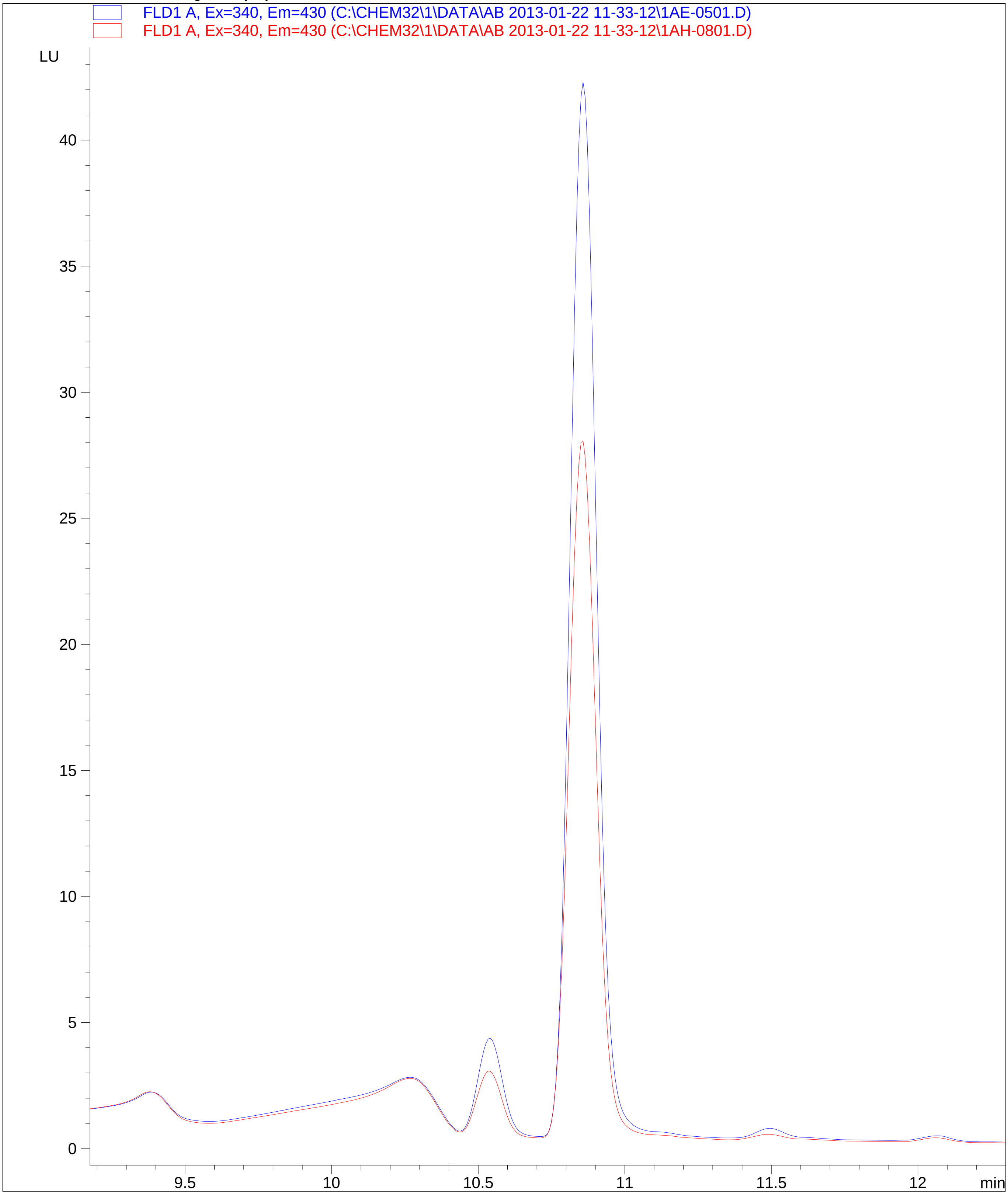

Supplement: S2 Fig — Tyramine (150 mg/L) was incubated with 2 μL of P. acidilactici CECT 5930 recombinant enzyme (28 μg) for 24 h, as described in Material and Methods section. Then, remaining tyramine concentration was determined by LC-FLD. The graph shows the retention time on the x-axis versus fluorescence units (LU) recorded by the detector (y-axis). Only the chromatographic profile region corresponding to the tyramine peak is shown. Note the peak area corresponding to incubation with recombinant enzyme (red) is approximately 35% lower than the control (blue). As negative controls, an identical mixture without enzyme (control 1), and with heat inactivated enzyme (control 2) were used. Since both controls gave the same peak area, only one is shown (blue). (PDF) [file pone.0186019.s002.pdf]
